# Multifunctional Motion Sensing Enabled by Laser-Induced Graphene

**DOI:** 10.3390/ma16196363

**Published:** 2023-09-22

**Authors:** Bowen Deng, Zongyuan Wang, Weiguang Liu, Bin Hu

**Affiliations:** School of Optics and Photonics, Beijing Institute of Technology, Beijing 100081, China; 3120220542@bit.edu.cn (B.D.); cnwangzongyuan@163.com (Z.W.); kissyliu@yeah.net (W.L.)

**Keywords:** laser-induced graphene, flexible sensor, motion monitoring

## Abstract

The development of flexible sensors based on laser-induced graphene (LIG) has recently attracted much attention. It was commonly generated by laser-ablating commercial polyimide (PI). However, the weak mechanical extensibility of PI limits the development and diversified applications of LIG-based sensors. In this work, we adopted medical polyurethane (PU) tapes to peel off the LIG generated on PI and developed flexible and wearable sensors based on the proposed LIG/PU composite structure. Compared with other methods for LIG transfer, PU tape has many advantages, including a simplified process and being less time-consuming. We characterized the LIG samples generated under different laser powers and analyzed the property differences introduced by the transfer operation. We then studied the impact of fabrication mode on the strain sensitivity of the LIG/PU and optimized the design of a LIG/PU-based strain sensor, which possessed a gauge factor (GF) of up to 263.6 in the strain range of 75–90%. In addition, we designed a capacitive pressure sensor for tactile sensing, which is composed of two LIG/PU composite structures and a PI space layer. These LIG flexible devices can be used for human motion monitoring and tactile perception in sports events. This work provides a simple, fast, and low-cost way for the preparation of multifunctional sensor systems with good performance, which has a broad application prospect in human motion monitoring.

## 1. Introduction

As one of the key structures of electronic skin [1], flexible sensors are equivalent to the receptors on the surface of human skin, which are widely used in the fields of soft robotics, biomedicine, and micro-wearable devices. Researchers have developed many types of flexible sensors to meet the application requirements in these fields. Wan et al. [2] developed an artificial synaptic thin-film transistor built on an ultra-thin flexible substrate based on high-carrier mobility semiconductor single-wall carbon nanotubes. The neuroelectronic skin closely mimics the behavior of actual human skin and can detect force stimuli in real time and provide behavior similar to biological synapses to transmit stimulus signals to the next stage. With the development of virtual reality technology and human–computer interaction technology, sensors with multifunctional application scenarios have attracted much attention. Qu et al. [3] developed a Ti3C2Tx-based strain sensor (GF = 19.1) in 2023 and used it for audio detection, human physiological signal monitoring, and facial expression recognition. To evaluate the performance of the proposed strain sensors, the gauge factor is adopted using the following formula:

(1) $$GF=\frac{\Delta R/R}{\varepsilon_{0}}$$

where Δ*R*/*R* is the relative resistance change of the strain sensor, and *ε*_0_ is the strain degree. At the same time, flexible sensors with different functions have been developed, including pressure sensors [4,5], temperature sensors [6,7], humidity sensors [8,9], and multifunctional integrated sensors [10] that can simultaneously respond to multiple external stimulus signals. They are widely used in medical monitoring, human-computer interaction, intelligent sensing, and other fields. However, most flexible sensors have shortcomings and drawbacks due to the electrode material itself. For example, the mechanical properties of the metal electrode [11] are weak, and the patterned structure depends on the micro-nano processing technology. Traditional graphene preparation methods are complex and high cost [12,13], making it difficult to quickly complete batch preparation. Simultaneously, some materials are harmful to skin and unsuitable for developing flexible human wearable devices. These limits are not conducive to the development of flexible sensing technology. Therefore, finding a low-cost, efficient way to prepare flexible sensors with high performance is significant.

In 2014, laser-induced graphene (LIG) technology was proposed [14]. Patterned 3D porous graphene structures can be directly fabricated using laser-sculpting polyimide (PI) films with good ductility and thermal and electrical conductivities. With the advantages of low cost and high speed, it simplifies the tedious process of traditional patterned graphene production, realizing one-step preparation. The good structure and properties of LIG have become the focus of researchers seeking to develop flexible sensors. Battina Sindhu et al. [15] developed an Internet of Things (IoT) sensor based on a LIG/PI patch antenna. The proposed sensor has a sensitivity of 14.08 and 11.34 to compressive strain and tensile strain, respectively, which can be used for motion detection, structural health monitoring, and industrial strain sensing applications. In addition, there are flexible sensors for strain and pressure detection [16], microsensors for sound detection and recognition [17], and electrochemical sensors for the biomedical field [18,19,20].

However, the poor mechanical properties of the PI base affect the flexibility of the sensors and limit the diversity of functions. Therefore, it is crucial to find a more flexible base material. In this regard, Li et al. [21] induced the generation of LIG on silk by a heat treatment and laser direct-writing, two-step method, studied the influence of laser parameters on the electrical conductivity of LIG, and developed a flexible sensor that can be used for strain monitoring, with a linear strain factor of 20.6 in the range of 0–30% strain. In addition, Wang et al. [22] creatively proposed a dual-mode liquid recognition strategy based on time and velocity to identify various liquids based on LIG paper. Compared to PI films, they have better flexibility but do not have good elasticity for stretching and other deformation. This limits the functions and application scenarios of flexible sensors. In addition, some researchers have found that polydimethylsiloxane (PDMS) can be used to transfer LIG from PI while the structure of LIG is not destroyed. Flexible sensors based on LIG/PDMS have been developed [23,24]. This approach usually involves three steps. First, the PDMS solution is prepared and then poured into the mold to obtain semi-cured PDMS. Then, LIG is extracted from the parent PI film by a simple laminating process. Finally, fully cured LIG/PDMS is obtained by high-temperature baking. Therefore, the transfer method of PI onto PDMS is complicated, which is not suitable for the efficient preparation of flexible sensors based on LIG.

Polyurethane (PU) is a non-toxic and eco-friendly material that is harmless to human skin and widely used in clothing fabrics, medical treatments, leather, and other fields. There are many micro-holes in the diameter of 10–50 μm in PU film [25], which can effectively pass through water vapor and block water droplets simultaneously to achieve good waterproof and breathable function. At the same time, polyurethane-dense structures can effectively isolate dust in the air. A medical PU tape has a high viscosity [25], high permeability, and ultra-thin performance, which makes it an ideal substrate material for flexible sensors. Ren’s group [25] used a medical PU tape (45 μm) to transfer LIG to develop an electrooculography and tactile perception collaborative interface for 3D human−machine interaction. This work proved that PU film has a great potential value as a substrate for LIG transfer.

Here, we used an ultra-thin medical PU tape (25 μm) to transfer LIG from a PI base. We systematically studied the structure and conductivity changes of LIG prepared under different laser powers before and after the transfer process, the sensitivities of LIG structures with different strain directions, and the influence of different pattern sizes on the strain sensitivity. We proposed a multifunctional, flexible sensing system for human sports monitoring. The system includes two modules: strain sensing and pressure sensing. A flexible strain sensor with high sensitivity (GF = 263.6 at high strain), high response speed (150 ms), and good stability (1000 cycles) is optimized and developed for human motion monitoring (such as gesture recognition, etc.). We inserted a very thin PI film into two layers of LIG/PU. The three layers are tightly fitted to form a plate capacitor, which can be used as a flexible pressure sensor for pressure sensing. At the same time, our sensors have the advantages of being ultra-thin, breathable, and waterproof with PU tape as the substrate. The research in this paper has great application potential in the fields of human motion monitoring and intelligent sensing in sports events.

## 2. Methods

### 2.1. Materials and Preparation

A commercial PI film (Kapton, Camalgori, China) with a thickness of 80 μm was attached to a quartz substrate (50 × 50 × 1 mm) for LIG preparation (ambient conditions). Conductive copper foil (5 mm wide) and silver paste (heat-cured type) were used for connecting sensor electrodes. Medical PU tape (25 μm) was chosen for the transfer and encapsulation of LIG. The micropore structure of PU can be observed under the electron microscope, as shown in Figure S1. The PI film was cut into an eligible size and tightly bonded onto the quartz. Then the surface was cleaned with anhydrous ethanol and dried naturally for laser induction. The laser cutting system (Nano Pro3, continuous wave laser, 450 nm) was controlled to scan in both the X- and Y-directions to complete the preparation of the LIG patterns. The diameter of the laser spot was 120 µm, and the laser output power was continuously tuned from 0 W to 5 W with a step of 0.05 W. The laser scanning speed was set to 10 cm/s.

**Figure 1:**
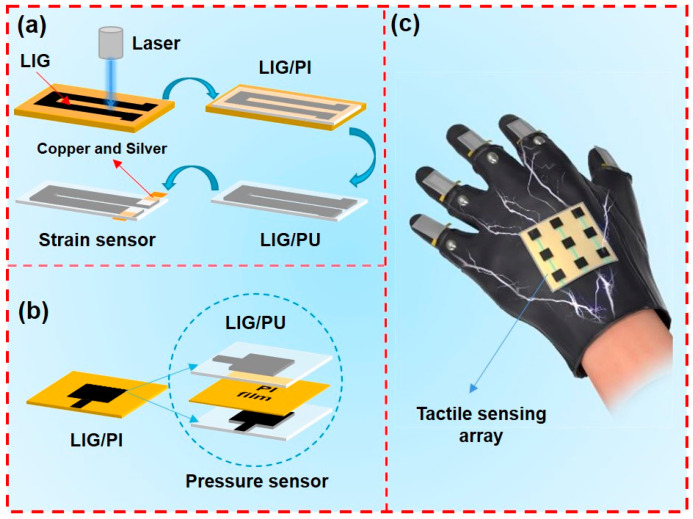
Schematic illustration of the fabrication of LIG/PU sensors. (**a**) The fabrication process of a LIG/PU strain sensor. (**b**) The fabrication process of a LIG/PU pressure sensor. (**c**) Diagram of the integrated sensors.

Based on the LIG/PU, we developed a system for human motion monitoring that is suitable for winter sports, including flexible strain sensors for strain detection and flexible pressure sensors for tactile feedback, which is demonstrated in Figure 1. After transferring a LIG pattern by using a PU film, we soldered the LIG sensors with copper foil and silver paste. The PU tape was used as the flexible base of the sensor; LIG was used as the sensing part; and copper foil was used as the electrode to achieve the measurement. Then the preparation of a LIG/PU strain sensor was completed, as shown in Figure 1a. Additionally, we used two 3 × 3 LIG arrays as electrode planes and a layer of PI film as the space layer to construct the tactile sensor. The tactile sensing array preparation was completed as depicted in Figure 1b. Finally, these devices were integrated into a glove, as demonstrated in Figure 1c.

### 2.2. Measurements and Characterizations

The surface morphology of the samples was characterized by a scanning electron microscope (SEM, SHIMADZU SSX-550, Kagoshima, Japan). The Raman scattering spectra were then characterized by a Raman spectrometer (RENISHAW RM2000, London, UK). The resistivity of LIG samples generated at different laser powers was tested on a four-probe measurement system (RTS-9, PROBES TECH, Beijing, China), while the resistance of LIG samples with different line widths was tested using a multimeter (PROSKIT MT-1280, China). The responses of LIG strain sensors and LIG pressure sensors were tested with a DM3058 digital multimeter (Rigol Technologies). A repeatability test of the sensors was performed by an electric stretching machine (LONG XIANG XWFTL, Beijing, China).

## 3. Results and Discussion

### 3.1. Structural Analysis and Characterization

**Figure 2:**
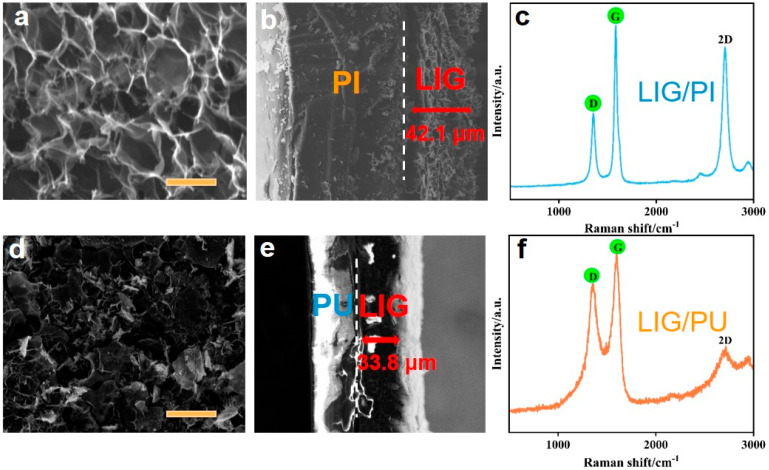
SEM images and Raman spectra of LIG/PI and LIG/PU samples. (**a**) SEM image of LIG/PI surface morphology (Scalar bar is 5 μm). (**b**) SEM image of LIG/PI cross-section. (**c**) Raman spectra of LIG/PI. (**d**) SEM image of LIG/PU surface morphology (Scalar bar is 20 μm). (**e**) SEM image of LIG/PU cross-section. (**f**) Raman spectra of LIG/PU.

We prepared the LIG/PI sample at 4.5 W (atmospheric conditions). Then, we characterized the surface morphology of LIG/PI using SEM. Figure 2a shows the SEM image of the LIG surface. The three-dimensional porous network structure can be clearly seen. Figure 2b depicts the cross-section SEM image of LIG/PI. It can be seen that there is a clear interface between LIG and PI, and the thickness of LIG is 42.1 μm. Previous studies [26] have shown that the oxygen extraction temperature of PI is 550 °C, the carbonization temperature is 700 °C, and the graphitization temperature is 3000 °C. The large amount of heat generated by laser causes a rapidly rising local temperature, which destroys the C-O, C=C, and N-C bonds and recombines the C atoms to form the graphene network [14]. The Raman spectrum of LIG prepared at 4.5 W is shown in Figure 2c. When the laser power exceeded the threshold for LIG formation, the D peak would appear at 1350 cm^−1^, the G peak appeared at 1590 cm^−1^, and the 2D peak appeared at 2700 cm^−1^, indicating the graphitization of PI and the formation of graphene [14]. The G peak was caused by in-plane vibrations of sp^2^ carbon atoms, while the D peak was caused by defects of graphene itself or the curved sp^2^ carbon bond (the curved graphene layer) [27].

Subsequently, we used PU tape to peel off LIG from PI. We first pasted PU tape on the surface of the LIG, then used an aluminum plate to cover the PU tape, and then a pressure meter was used to press the aluminum plate and maintain a fixed pressure for 20 s. Due to the high adhesion of PU tape, LIG can be effectively peeled and attached to PU tape. As shown in Figure 2d, the SEM image shows that the LIG transferred onto the PU tape still presents a three-dimensional porous structure without local rupture, indicating guaranteed quality. Figure 2e illustrates the cross-section SEM image of LIG/PU, which indicates the thickness of the LIG was 33.8 μm, and the thickness of the PU film was 25 μm. Compared with LIG/PI, the thickness of the LIG became smaller because it was pressed during the transfer option. The Raman spectrum of LIG/PU shows that the three typical graphene-characteristic peaks remained at 1351 cm^−1^, 1590 cm^−1^, and 2700 cm^−1^, corresponding to the D peak, the G peak, and the 2D peak, respectively (Figure 2f). The prominent D-peak indicates the presence of rich interfaces and disordered edges in graphene, which can be visually confirmed by the SEM image of Figure 2d. The I_G_/I_D_ ratio in the LIG Raman after transfer was lower than that before transfer, suggesting a decrease in the purity of LIG [28] during compression transfer, which could be expected due to the strong viscous substance of PU tape penetrating into LIG. However, it is noticeable that the 2D peak becomes weak in LIG/PU. This phenomenon can be attributed to the fact that the bottom LIG layer in the LIG/PI structure became the outermost layer of LIG/PU structure after the transfer operation. Because the middle region of PI received less laser heat than that on the PI surface, it can be inferred that the bottom LIG layer in LIG/PI had worse quality. Therefore, the surface properties of LIG on LIG/PU were worse than those on the surface of LIG/PI. At the same time, the surface of the transferred LIG may appear uneven due to the influence of the operation. In addition, the ultra-thin and soft characteristics of the PU substrate make it impossible to stick onto the test platform smoothly, thus causing some noise scattering in the test signal. This phenomenon increases the noise scattering in Raman testing, leading to a decrease in the signal-to-noise ratio.

### 3.2. Resistance of LIG/PI and LIG/PU

**Figure 3:**
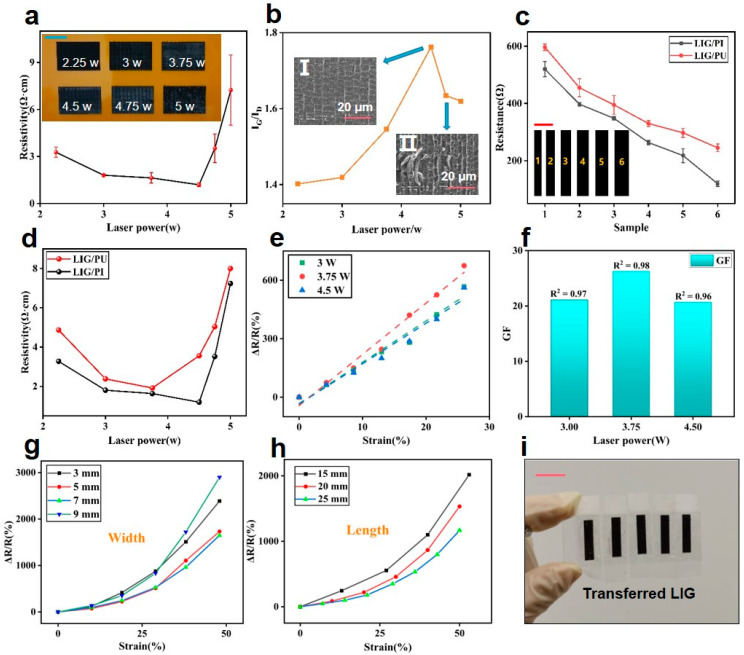
The electrical characteristic of LIG before and after transfer. (**a**) Photographs of LIG/PI samples generated at 6 different laser powers and their resistivity (Scalar bar is 1 cm). (**b**) Ratio of I_G_ to I_D_. The insets depict SEM images of LIG on PI film generated at laser powers of 4.5 W and 4.75 W, respectively. (**c**) Resistance of LIG/PI and LIG/PU with different widths (Scalar bar is 5 mm). (**d**) Resistivity of LIG/PI and LIG/PU generated at different laser powers. (**e**) Resistance response of LIG/PU generated at different laser powers, dependent on strain changes. (**f**) The gauge factors (GFs) of LIG/PU samples. Resistance response of LIG/PU with different widths (**g**) and lengths (**h**), dependent on strain changes. (**i**) Photograph of LIG/PU after transfer (Scalar bar is 1 cm). From left to right: LIG/PU under the laser power of 2.25 W, 3 W, 3.75 W, 4.5 W, and 4.75 W, respectively.

We first studied the influence of different fabrication laser powers on the quality of LIG/PI and LIG/PU, and then carried out Raman spectra analysis and resistivity testing. Different laser powers (2.25 W, 3 W, 3.75 W, 4.5 W, 4.75 W, and 5 W) were set to generate LIG samples. The sizes of the six LIG samples are all 1 × 2 cm. The laser luminous flux can be calculated using the following formula [29]:

(2) $$\phi =\frac{P}{v\cdot d}$$

where *Φ* is the luminous flux, *P* is the laser power, *v* is the scanning speed, and *d* = 120 μm is the diameter of the laser spot. As the scanning speed was set to 10 cm/s, the corresponding laser flux under the minimum power used in this work (2.25 W) was 18.75 J/cm^2^, which is larger than the threshold to generate LIG (~5.5 J/cm^2^) reported in reference [26]. The four-probe measuring system was used to measure the resistivity of LIG samples. The results are shown in Figure 3a. It indicates that the resistivity of LIG presents a trend of decline with the gradual increase of power within a certain range. The minimum resistivity (1.145 Ω·cm) of LIG appears at the power of 4.5 W. However, when the power continues to increase, the resistivity presents an increasing trend. More SEM images of LIG generated at different laser powers can be found in Figure S2. With the increase in laser power, the porosity of the internal structure of LIG was increased, which expanded the surface area of its conductive network, finally improving its conductivity. The intensity ratio of the G peak and the D peak (I_G_/I_D_) is commonly used to evaluate the quality of LIG. (Raman spectra of LIG/PI samples obtained at different laser powers are shown in Figure S3). Figure 3b shows the relationship between I_G_/I_D_ and laser power. The increasing I_G_/I_D_ indicates that the purity of LIG is improving [28]. When the power increased to 4.75 W, I_G_/I_D_ began to decline. The insets in Figure 3b illustrate the corresponding SEM images of LIG generated at the power of 4.5 W and 4.75 W, respectively. It indicates that LIG would be damaged when the power was excessive (4.75 W), which also caused a certain degree of decrease in its electrical conductivity (Figure 3a).

Based on the above research, the laser power of 4.5 W and scanning speed of 10 cm/s were selected for the preparation of the LIG electrode, thus the LIG with the highest conductivity was obtained. We then tested the LIG single-wire resistors with different widths. Single-line patterns with various widths (1.88 mm, 2.35 mm, 2.82 mm, 3.29 mm, 3.76 mm, 4.23 mm, and 4.7 mm) were printed, as shown in the inset of Figure 3c. The multimeter was used to measure the resistance at both ends, and the results before and after transfer are shown in Figure 3c. It can be seen that the resistance of LIG decreased in a linear trend as the width increased. Additionally, the resistance of all LIG/PU samples are higher than LIG/PI samples. To explain this phenomenon, LIG could be regarded as a regular conductor, whose resistance *R* can be given by:

(3) $$R=\frac{\rho \cdot L}{S}$$

in which *ρ* is the resistivity of LIG, *L* is the length of LIG, and *S* is the cross-sectional area of the LIG. It is known that when the laser parameters are fixed, the thickness of the generated LIG remains roughly constant [14]. As the width increases, the corresponding cross-sectional area increases, contributing to the decreased resistance. This indicates that the LIG prepared in this method has good uniformity and stability.

It is noticeable that the resistance was changed after transfer, which was probably due to the resistivity change. We thus tested the resistance of the LIG/PU. Figure 3d shows the comparison of the resistivities of LIG/PU and LIG/PI. It can be seen that the variation trend of LIG/PU resistivity with laser power was similar to that of LIG/PI. At the same time, the resistivity of LIG/PU increased slightly compared to that of LIG/PI, which can be attributed to the process of transferring LIG using PU tapes. When we used PU tape to paste the LIG in the transfer, the operation of pressing would break the porous structure of the LIG and cause some unsmooth areas due to adhesion, resulting in a decrease in the resistivity of the graphene. Although the conductivity of the LIG after transfer was reduced, the ductility and strain range of the material were improved, which was conducive to the preparation of strain sensors with higher response ranges.

Subsequently, we tested the strain response of LIG/PU prepared under 3 laser powers, as shown in Figure 3e. The relative resistance maintained a good linear response with strain. Figure 3f shows the GF obtained by fitting the curves in Figure 3e, demonstrating the GF of LIG/PU at these 3 powers was similar, which may be attributed to the fact that differences in the 3 LIG structures caused by the compression process were small. Strain sensitivity tests of LIG/PU with different line widths and lengths were performed, which are shown in Figure 3g,h, respectively. It was found that with an increase in width or length, the change in LIG/PU resistance became smaller under the same strain. After the LIG was transferred to the PU tape, it was bound tightly to the PU due to the extremely high viscosity. Thus, when a small strain was applied, LIG was constrained by PU and changed with the whole PU film. Therefore, it can be inferred that when the same strain is applied, the change in size of LIG/PU is fixed. In this case, the GF is inversely proportional to the initial LIG resistance. Figure 3i shows the photographs of LIG/PU after the transfer, which confirms that PU is suitable for transferring LIG effectively with high quality.

### 3.3. Performance of Strain Sensor

**Figure 4:**
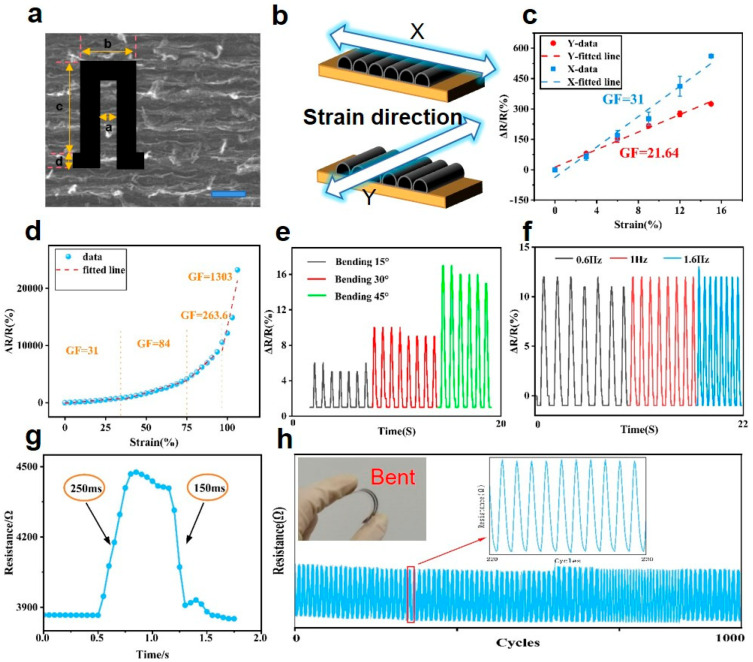
Mechanical and electrical performance of the LIG/PU strain sensor. (**a**) SEM image of LIG (Scalar bar is 50 μm) and the parameters of LIG electrode pattern (a = 5 mm; b = 15 mm; c = 30 mm; and d = 3 mm). (**b**) Diagram of applying strain in different directions. (**c**) Resistance response of the sensor with strain applied along X and Y directions, respectively. (**d**) Resistance response of the sensor at a strain of 0–100% in the X direction. (**e**) Relative resistance changes under different bending angles of 15°, 30°, and 45°. (**f**) Relative resistance changes under different frequencies of 0.6, 1.0, and 1.6 Hz. (**g**) Time domain response of resistance changes under stretching. (**h**) Bending stability under 1000 cycles.

Based on the previous study of resistance characteristics, we prepared the LIG/PU sensor at a laser power of 3.75 W. Microscopic observation revealed that the LIG exhibited an anisotropic nature in the X and Y directions, resulting in a gully-like texture on its surface, as shown in Figure 4a. The LIG sensing pattern is illustrated as the inset in Figure 4a, with the parameters: a = 5 mm; b = 15 mm; c = 30 mm; and d = 3 mm. The strain sensitivity of the sensor was tested in these two mutually perpendicular directions, as shown in Figure 4b, and the results are presented in Figure 4c. It can be seen that the GF in the X direction was significantly higher than that in the Y direction. This phenomenon can be explained by the surface structure of the LIG. The scanning mode of the laser was progressive scanning row by row along the X direction, which eventually led to the fringe-like surface structure of LIG. As a result, when the strain was applied in the direction of X, the connection between the graphene fringes was more affected, leading to a larger increase in resistance and higher GF. The strain sensitivity curve of our sensor is shown in Figure 4d. The experimental results indicate that the effective sensing range of the sensor was 0–100%, with a GF of 23.4 at 0–32% and 84 at 32–75%. The GF was as high as 263.6 in the range of 75–90% and 1303 in the range of 90–100% under high strain conditions, confirming that LIG/PU has a high sensitivity that is significantly superior to other flexible strain sensors (Table 1). Figure S4 shows microscopic images of the LIG surface structures under different tensile strains. When a small tensile strain was applied, the internal structure of LIG became loose but still connected, and the resistance rose slightly. When the strain degree increased further, the surface structure of the LIG appeared to locally fracture, which greatly increased the total resistance.

Additionally, we conducted further research on sensor recoverability and stability, as demonstrated in Figure 4e. The stable circulation curves of the sensor at 15°, 30°, and 45° bending angles indicated good repeatability. As shown in Figure 4f, resistance changes were measured at frequencies of 0.8, 1.0, and 1.6 Hz, respectively. The results confirm that there was no obvious resistance change at different stretching frequencies. The independent frequency characteristic of strain sensors is crucial for the stability and reliability of signal detection. Figure 4g shows the response time of the sensor, with a strain application time of 250 ms and a strain removal time of 150 ms. A device life test of 1000 cycles at 45° bending strain confirmed good durability of the sensor, as shown in Figure 4h. The experimental instrument and partial results of this test are shown in the Figure S5.

### 3.4. Performance of the Pressure Sensor

**Figure 5:**
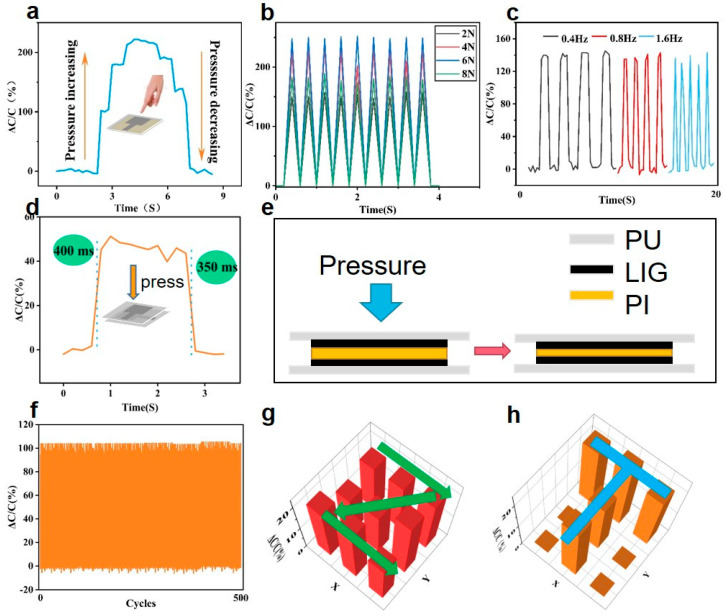
Mechanical and electrical performance of the LIG/PU pressure sensor. (**a**) Recoverability under stepped pressure. (**b**) Dynamic responses under repeated pressure. (**c**) Relative capacitance changes at different frequencies. (**d**) Dynamic response of relative capacitance changes under pressure. (**e**) Schematic diagram of working principle of pressure sensor. (**f**) Stability of the pressure sensor under 500 cycles. Relative capacitance changes of tactile sensors when the finger slid along the tactile sensing array along “Z” path (**g**) and “T” path (**h**).

The flexible pressure sensor was then fabricated, consisting of the unit structure shown in Figure 1b. Subsequent capacitance signals were obtained using DM3058. The capacitance of a parallel plate capacitor can be given by the following formula:

(4) $$C=\frac{\varepsilon S}{4\pi kd}$$

where *ε* is the dielectric constant of the PI film, *S* is the electrode area, and *d* is the distance between the two electrodes. Deformation caused by pressure on the PU film would result in a change in capacitance. Figure 5a shows the response of the relative capacitance change as a function of fingertip pressure, which indicates a good recoverable ability. The repeatability of the sensor under different pressures (2, 4, 6, 8 N) and frequencies (0.4, 0.8, 1.6 Hz) was then investigated, as shown in Figure 5b,c, respectively. The results demonstrated the good repeatability of the pressure sensor. The response time of the sensor was measured at 350 ms, as shown in Figure 5d. The cross-section diagram and working principle of the pressure sensor are shown in Figure 5e. Figure 5f depicts the relative capacitance change of the pressure sensor, demonstrating considerable consistency without obvious fatigue during 500 cycles. Photographs of the tactile sensor and the capacitive response of the 9 units of the pressure-sensing array are shown in Figure S6. We finally tested the respective performances of the tactile sensors when a finger slid along a “Z”- shaped path (Figure 5g), and placed a “T”-shaped mold (Figure 5h) on the sensing array. Real-time pressure distributions were successfully obtained, which demonstrated effective feedback.

### 3.5. Motion Sensing System

**Figure 6:**
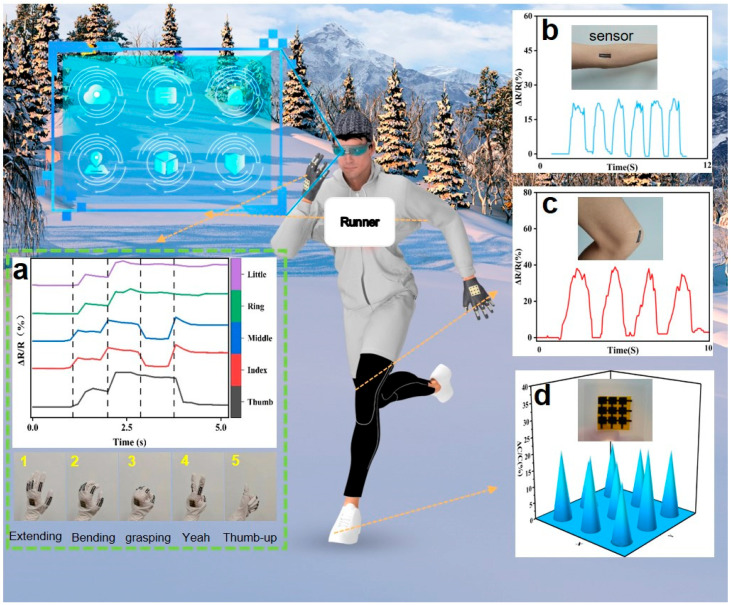
Schematic diagram of the motion sensing system. (**a**) Gesture recognition. (**b**) Elbow joint motion detection. (**c**) Knee joint motion detection. (**d**) Measurement of pressure distribution in the sole.

Integrating flexible sensors based on LIG/PU allows us to monitor human motion status comprehensively. The DM3058 digital multimeter can measure changing electrical parameters in real time and store the data. Thus, we can get the dynamic data of the measurement by connecting the sensor to the device. We attached five strain sensors to five fingers, respectively, which could effectively monitor the changes in human gestures, as shown in Figure 6a. The resistance-response curves of the five strain sensors were different under various gestures. According to the relative resistance of different sensors, gesture recognition was realized. This device can be combined with augmented reality (AR) glasses to control virtual interfaces with gestures in the future. By attaching sensors to the elbow and knee joints of the human body, changes in posture during sports such as running can be monitored. Figure 6b,c shows the measurement results. The responding signal strength increases with an increase in bending angle, showing a stable and repeatable responding signal. Flexible pressure sensors based on LIG/PU can be used for tactile feedback. Attaching a 3 × 3 sensor array to the sole of a human foot allows the pressure distribution in motion to be monitored. Figure 6d shows the relative capacitance distribution of the tactile sensor array. The LIG/PU sensor shows good stability and repeatability in all of the above signal monitoring.

## 4. Conclusions

In this study, we employed PU tapes for the transfer of LIG from PI. Our research focused on comprehensively investigating the structural and material properties of LIG/PI and LIG/PU. We also analyzed the influence of different laser powers and LIG linewidths on the performance of the strain sensor. Based on these investigations, we successfully developed flexible sensors using LIG/PU composites, including strain and pressure sensors. The production process of these sensors is characterized by its simplicity and efficiency. The flexible strain sensor we designed is ultra-thin, waterproof, and transparent, exhibiting remarkable strain sensitivity in the strain range of 0–100%. It provides accurate strain measurements with a GF of 263.6 at 75–90%. Additionally, we developed a capacitive pressure sensor array suitable for tactile perception, enabling reliable feedback on pressure distribution. This array is particularly useful in analyzing athletes’ running postures. By combining these devices, we established a comprehensive system for monitoring human motion. Furthermore, the integration of this system with AR equipment provides users with a method to control virtual interfaces enabled by gesture recognition. The pressure sensing array, incorporated into footwear, offers real-time feedback on pressure distribution, facilitating detailed analysis of athletes’ running mechanics.

Overall, our work presents a simple, fast, and cost-effective method for developing flexible sensing equipment for monitoring human motion. The findings of this study have implications for the field and open up possibilities for various applications.

## Figures and Tables

**Table 1:** Performance comparison between our strain sensor and the reported strain sensors.

Substrate	Electrode Materials	Sensing Range	GF	Ref
PI	LIG	0–3.3%	0.2057	[16]
CA/TPU	rGO	0.5–10%	3.006	[30]
PB	CB/CNT	0–0.7%	7.5	[31]
Ecoflex	PCNFs	0.1–11%	0.73	[32]
PDMS	Ti3C2Tx	0–50%	45	[33]
PI	Cu	0–35%	2	[34]
ORC	CB	0–46%	16	[35]
polyacrylamide	Acrylate sodium	0–300%	6.77	[36]
PDA/LC	PANI/rGO	0.2–50%	24	[37]
**PU**	**LIG**	**0–100%**	**263.6**	**This work**

## Data Availability

Data is available on request from the corresponding author.

## Supplementary Materials

The following supporting information can be downloaded at: https://www.mdpi.com/article/10.3390/ma16196363/s1, Figure S1: Characteristics of the PU film; Figure S2: SEM images of surface (left column) and cross section (right column) of LIG prepared on PI thin films at different laser powers before transfer; Figure S3: Raman spectra of LIG before transfer prepared at different laser powers; Figure S4: Microscopic images of LIG surface under different tensile strains; Figure S5: The instrument of bending cycle test for strain sensor and part of the bending cycle test results; Figure S6: Left: Model of the pressure sensing array. Right: The capacitance responding curve of each cell.
