# Supplementary material for: Multifunctional Motion Sensing Enabled by Laser-Induced Graphene
Source: Materials (Basel). 2023 Sep 22;16(19):6363. doi: 10.3390/ma16196363 (PMC10573838; doi:10.3390/ma16196363)
Supplement: Supplementary file 1 [file materials-16-06363-s001.zip › materials-2596947-supplementary.pdf]

Supporting Information

Multifunctional Motion Sensing Enabled by Laser-Induced Graphene

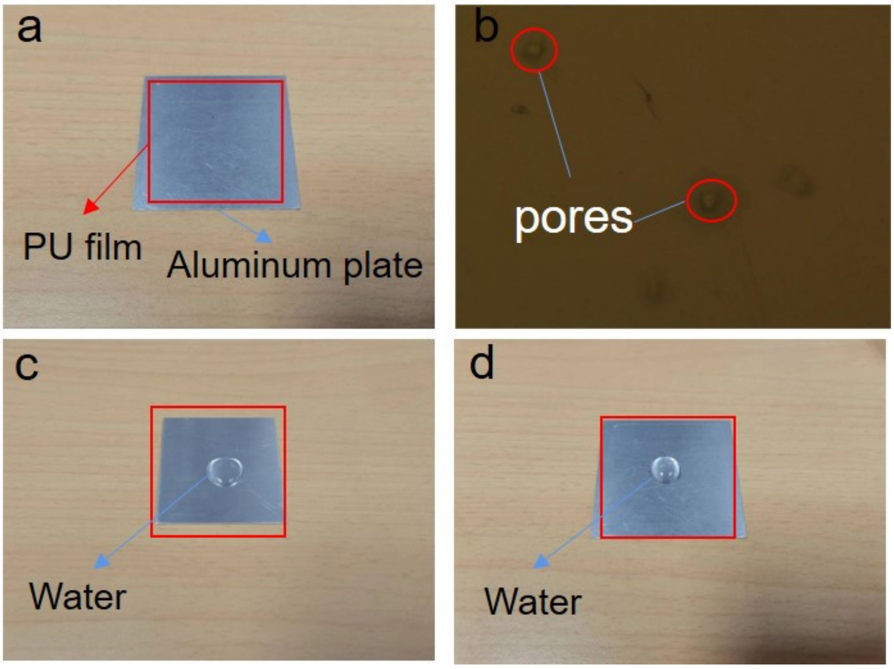

Figure S1. Characteristics of the PU film. (a) Transparency of PU film. (b) Image of PU film under microscope. (c) A drop of water on the aluminum plate. (d) A drop of water on the PU film pasted onto the aluminum plate.

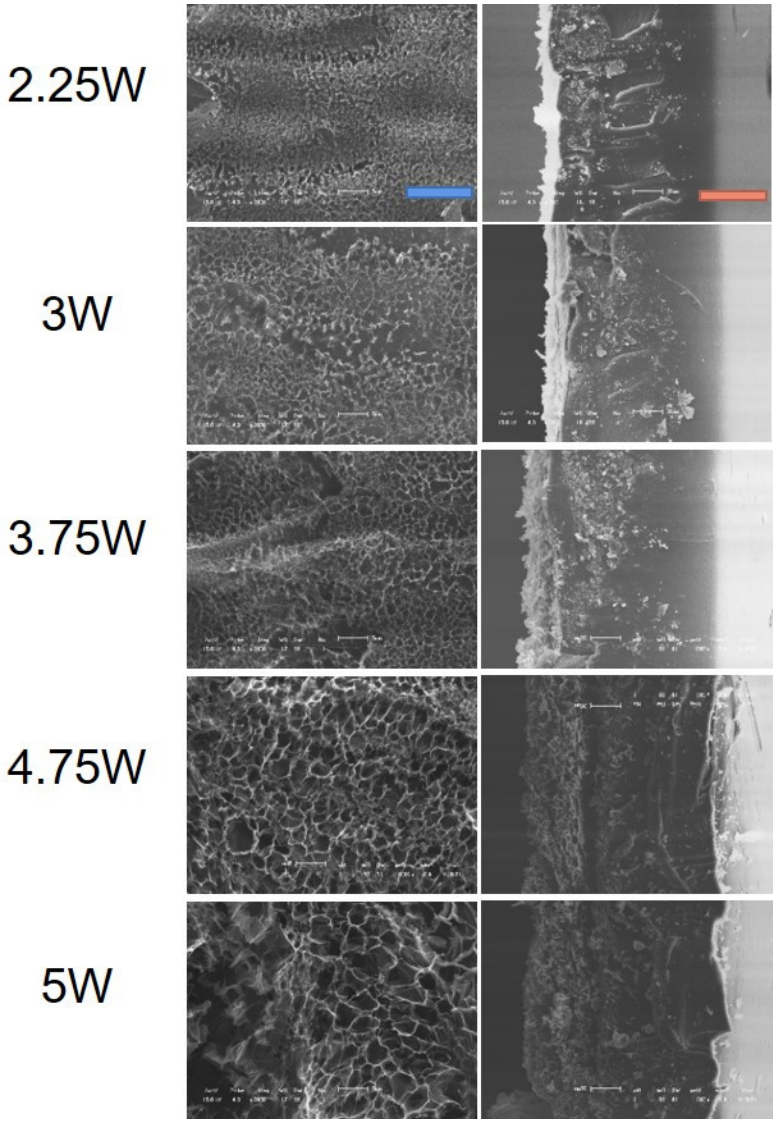

Figure S2. SEM images of surface (left column) and cross section (right column) of LIG prepared on PI thin films at different laser powers before transfer. (The blue and orange scalar bar are 5 μm and 20 μm, respectively.)

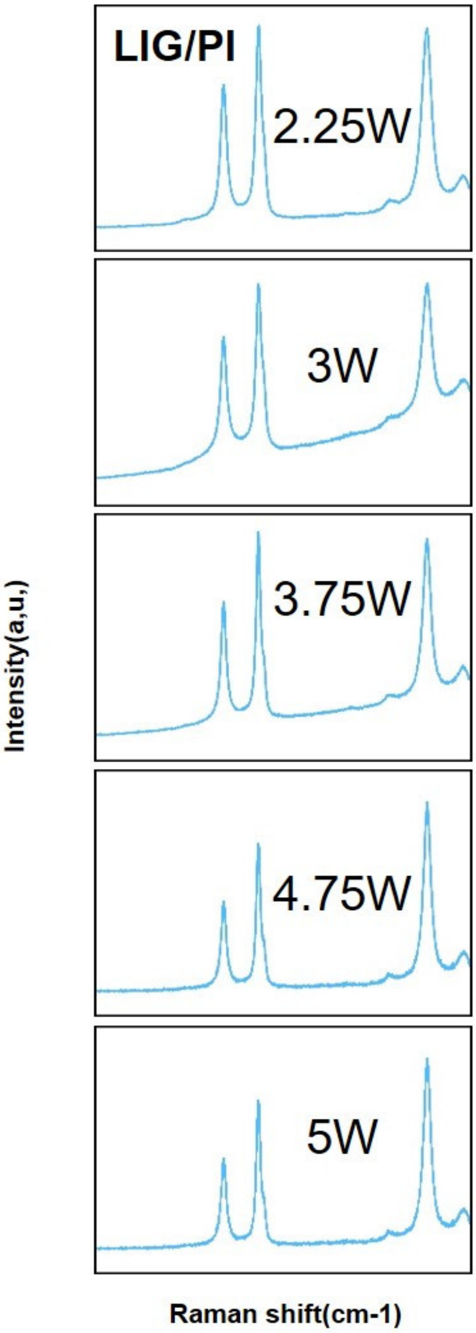

Figure S3. Raman spectra of LIG before transfer prepared at different laser powers.

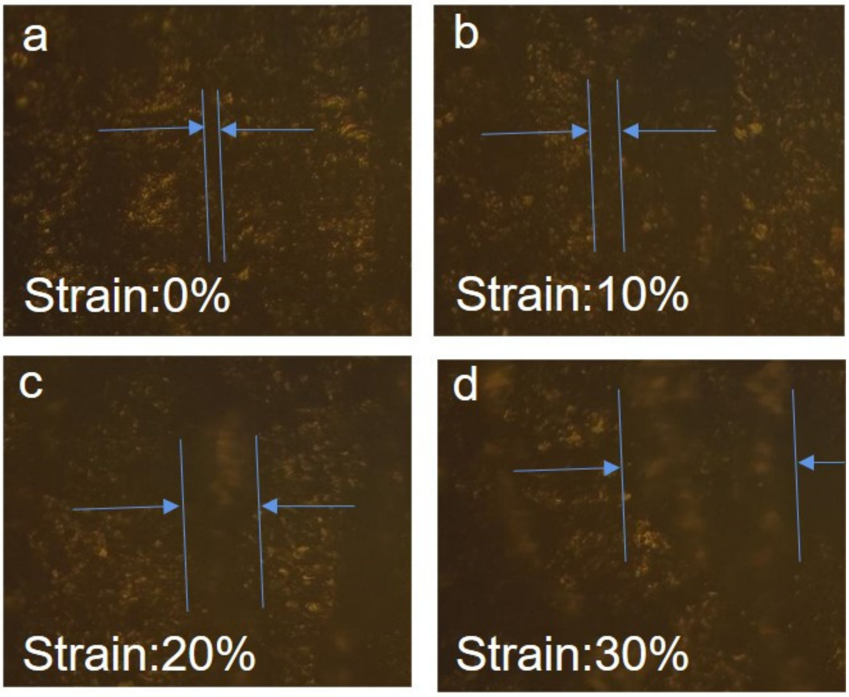

Figure S4. Microscopic images of LIG surface under different tensile strains.(a) Strain at 0%. (b) Strain at 10%. (c) Strain at 20%. (d) Strain at 30%.

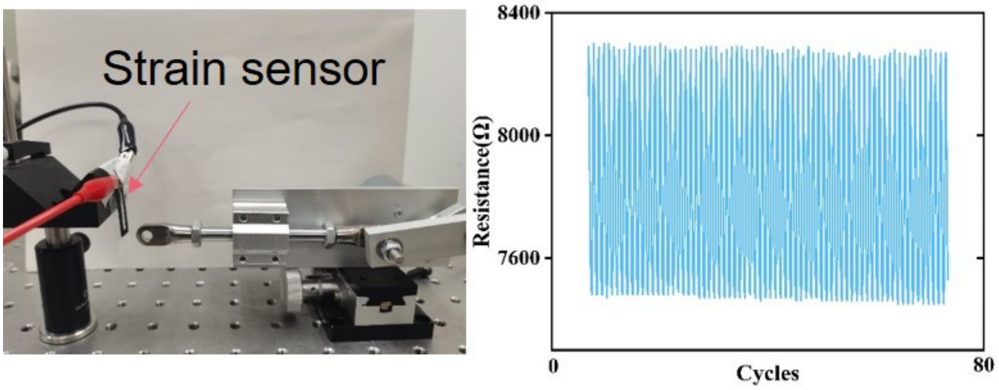

Figure S5. The instrument of bending cycle test for strain sensor and part of the bending cycle test results.

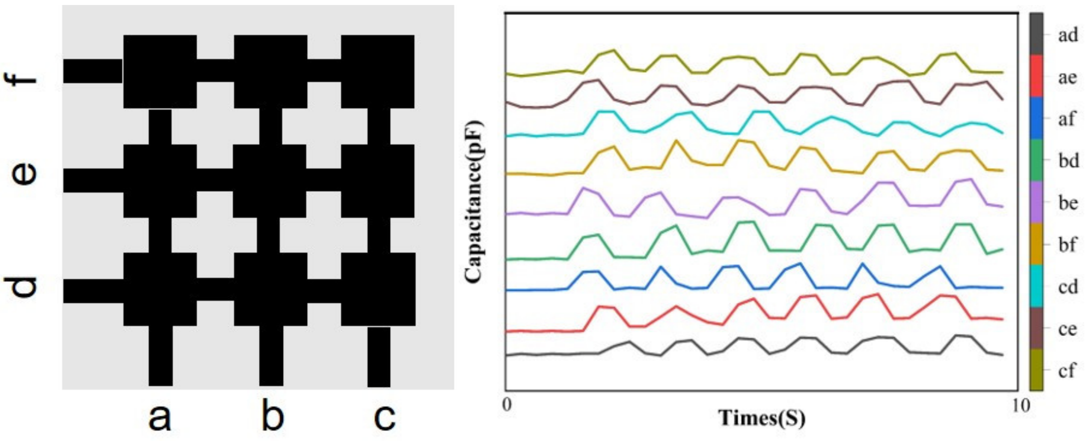

Figure S6. Left: Model of the pressure sensing array. Right: The capacitance responding curve of each cell.
